# Identification and Management Strategies for Intracoronary High Thrombus Burden in Patients With STEMI: A Practical Experience and Literature Review

**DOI:** 10.31083/RCM37466

**Published:** 2025-07-30

**Authors:** Xing Feng, Tongku Liu

**Affiliations:** ^1^Department of Cardiology, Jilin People’s Hospital, 132001 Jilin, Jilin, China; ^2^The Center of Cardiology, Affiliated Hospital of Beihua University, 132011 Jilin, Jilin, China

**Keywords:** ST-segment elevation myocardial infarction, coronary artery, thrombus burden, identification, management strategy

## Abstract

Acute myocardial infarction (AMI) includes ST-segment elevation myocardial infarction (STEMI) and non-ST-segment elevation myocardial infarction (NSTEMI). STEMI is the most severe type of AMI and is a life-threatening disease. The onset and progress of STEMI are accompanied by thrombosis in coronary arteries, which leads to the occlusion of coronary vessels. The main pathogenesis of STEMI is the presence of unstable atherosclerotic plaques (vulnerable plaques) in the vessel wall of the coronary arteries. The vulnerable plaques may rupture, initiating a cascade of blood coagulation, ultimately leading to the formation and progression of thrombus. Treating STEMI patients with high thrombus burden is a challenging problem in the field of percutaneous coronary intervention (PCI). During the PCI procedure, the thrombus may be squeezed and dislodged, leading to a distal embolism in the infarction-related artery (IRA), resulting in slow blood flow (slow flow) or no blood flow (no reflow), which can enlarge the ischemic necrosis area of myocardial infarction, aggravate myocardial damage, endanger the life of the patient, and lead to PCI failure. Identifying and treating high thrombus burden in the IRA has been a subject of debate and is currently a focal point in research. Clinical strategies such as the use of thrombus aspiration catheters and antiplatelet agents (platelet glycoprotein IIb/IIIa receptor inhibitors, such as tirofiban), as well as the importance of early intervention to prevent complications, such as no reflow and in-stent thrombosis, are highlighted in recent studies. Thrombus aspiration is an effective therapeutic approach for removing intracoronary thrombus, thereby decreasing the incidence of slow flow/no reflow phenomena and enhancing myocardial tissue perfusion, ultimately benefiting from protecting heart function and improving the prognosis of STEMI patients. Notably, deferred stenting benefits STEMI patients with high thrombus burden and hemodynamic instability. Meanwhile, antithrombotic and thrombolytic agents serve as adjuvant therapies alongside PCI. Primary PCI and stenting are reasonable for patients with low intracoronary thrombus burden. The article describes the practical experience of the author and includes a literature review that details the research progress in identifying and managing STEMI patients with intracoronary high thrombus burden, and provides valuable insights into managing patients with high thrombus burden in coronary arteries. Finally, this article serves as a reference for clinicians.

## 1. Introduction

Acute coronary syndrome (ACS) includes ST-segment elevation myocardial infarction (STEMI) and non-ST-segment elevation myocardial infarction (NSTEMI) based on electrocardiogram (ECG) findings. The pathogenesis and clinical manifestations of STEMI and NSTEMI differ, meaning the clinical management methods differ. The pathogenesis of STEMI involves an acute and complete coronary occlusion due to coronary plaque rupture, platelet aggregation, and thrombosis. In contrast, NSTEMI is characterized by unstable plaques that may result in slow occlusion of the coronary with or without thrombosis. The manifestations of STEMI often include ST-segment elevation on an ECG, while NSTEMI presents with ST-segment depression or T wave inversion without significant ST-segment elevation [1]. Therefore, the clinical management approaches are accordingly different. In particular, there has long been a controversy regarding the identification and management of STEMI patients with a high thrombus burden in the coronary artery [1]. The research progress in the past decade has shown that intracoronary thrombus aspiration is an effective measure to remove thrombi, which may reduce the incidence rate of slow blood flow (slow flow) and no blood reflow (no reflow) events, improve the perfusion of myocardial tissue level, and is beneficial to protect heart function and improve the prognosis of STEMI patients. Indeed, STEMI patients with an intracoronary high thrombus burden and/or hemodynamic instability can benefit mostly from deferred stenting [2, 3]. Antiplatelet and thrombolytic drugs can be used as an adjunct to percutaneous coronary intervention (PCI). For STEMI patients with lower coronary thrombosis burden, it is reasonable to recommend primary stenting. This article describes the practical experience of the author alongside a literature review, details the research progress of identifying and managing intracoronary high thrombus burden in STEMI patients, and provides valuable insights for clinical practitioners.

## 2. Pathogenesis of STEMI

The recent study results have demonstrated that most STEMI cases are caused by unstable plaque in the vessels of coronary atherosclerosis [3, 4, 5, 6, 7, 8]. Autopsy and intravascular studies *in vivo* have confirmed that fibroatheromas (FAs), especially thin-cap fibroatheromas (TCFAs), are the most common vulnerable lesions. TCFAs are prevalent in human coronary atherosclerotic lesions, including asymptomatic individuals and patients with stable and unstable angina pectoris [9, 10, 11]. Plaque rupture, collagen fibers, and lipids within the plaque are exposed, come into contact with the formed elements of the blood, and trigger the coagulation system, resulting in acute thrombosis within the coronary artery. Intracoronary thrombosis may block coronary blood flow, cause acute ischemic injury to the myocardium, and manifest as ST-segment elevation on the ECG. Persistent myocardial ischemia can lead to ischemic necrosis in the myocardium and pathological Q waves on the ECG. The time from coronary artery occlusion to the appearance of an elevated ST-segment on the ECG depends on the compensatory mechanisms involved in the pathophysiology and the speed of vascular occlusion. Generally, in the absence of collateral circulation, retrograde perfusion compensation, ST-segment elevation in the horizontal or upward type occurs when the coronary vessel is blocked for one minute. There is a time process from the onset of chest pain to an elevated ST-segment on the ECG, which depends on the speed of coronary vessel occlusion. Clinically, when we perform PCI treatment, and dilate the stenotic coronary artery with a balloon, the ST-segment elevation can be observed on an ECG following ballooning for 20 seconds (that is, the blood flow in the coronary artery is blocked for 20 seconds, and the ST-segment elevation may occur); the longer the blood flow is blocked, the higher the ST-segment elevations.

Persistent coronary artery spasms can also cause the onset of STEMI. Coronary spasms often occur in the vessel segments in coronary artery lesions and stenosis, and in the normal coronary artery vessel segments. While many factors can trigger coronary artery spasms, endothelial cell dysfunction is the main factor. The procoagulant substances released by activated platelets, including adenosine diphosphate (ADP), thromboxane A2, 5-hydroxytryptamine, platelet-derived growth factor, acetylcholine, etc., can cause endothelium-dependent relaxation in normal coronary artery vascular endothelial cells [12, 13, 14]. Endothelial cell dysfunction weakens the endothelium-dependent vasodilation effect, which may convert into a vasoconstriction effect. Under pathological conditions, endothelial cell activation increases vasoconstrictive substances, such as endothelin-1 and angiotensin II, thereby inducing coronary artery spasms, which can further aggravate endothelial cell damage, accelerate the development of coronary atherosclerosis plaques, further aggravate coronary artery stenosis, trigger the rupture of unstable plaques (vulnerable plaques), and result in acute myocardial infarction (AMI). The rupture of vulnerable plaques in the vessel wall of coronary atherosclerosis is related to atherosclerotic plaques, plaque structure, and the geometry of the coronary vessel lumen. Endothelial injury and plaque rupture expose the collagen fibers beneath the intima, which promotes rapid platelet adhesion, aggregation, and thrombosis, and activates the platelet membrane glycoprotein IIb/IIIa (GP IIb/IIIa) receptor, leading to the formation of platelet aggregate substance and thrombi [15, 16].

Inflammatory processes play an important role in the occurrence and development of acute coronary syndrome (ACS). Inflammatory factors participate in the occurrence of coronary atherosclerosis and the formation of unstable plaques [17, 18]. The pathophysiology of ACS includes four mechanisms: (1) Plaque rupture with inflammatory cell infiltration, which is the main pathological basis of ACS. This is usually local inflammation (such as monocyte infiltration) and systemic inflammation (the blood C-reactive protein (CRP) level is elevated in the patient). It is recommended to detect high-sensitivity C-reactive protein for patients with ACS; (2) plaque rupture does not necessarily occur with inflammatory cell infiltration. In certain instances, plaque rupture can exacerbate atherosclerosis, while some atherosclerotic plaques lack a significant presence of macrophages and do not exhibit elevated circulating CRP levels. Plaque rupture usually leads to a red thrombus rich in fibrin forming; (3) plaque erosion, one of the main causes of ACS, often leads to NSTEMI. Thrombi covering intimal erosion plaques are generally characterized as white structures rich in platelets; (4) coronary artery spasm may also cause ACS, which was previously considered a phenomenon of the epicardial coronary arteries, but in fact, also affects the coronary microcirculation. In the pathogenesis of ACS, monocytes and macrophages infiltrate and phagocytose low-density lipoprotein cholesterol (LDL-c) within the plaques, forming foam cells, which accumulate to form unstable plaques and lead to plaque rupture. Nonetheless, inflammatory cell infiltration may be absent in the lesions associated with plaque erosion [19, 20]. Recent research shows that cholesterol crystals (CCs) are a pathological pathway to plaque rupture [20, 21]. The evolution of vulnerable plaques is characterized by inflammation and physical changes. The LDL-c accumulated beneath the intima of vessels is phagocytosed by macrophages, which is transformed into esterified cholesterol (ESC). Cholesterol ester hydrolase converts ESC into free cholesterol (FRC) [20, 22]. Membrane-bound cholesterol carriers transport FRC to high-density lipoprotein (HDL). When the transport function of HDL is impaired and its composition is altered, FRC accumulates in the extracellular space. The reduced activity of cell membrane carriers can result in FRC accumulation within the cells. FRC saturation can lead to CCs forming, which may result in cell death and damage the endomembrane system [20, 23]. The imbalance between ESC and FRC will affect the formation of foam cells and CCs, which lead to the production of CRPs and interleukin-1β through the nucleotide oligomerization domain (NOD)-like receptor (NLR) family pyrin domain-containing protein 3 (NLRP3) inflammasome, which initiates the inflammatory reaction to destroy the stability of plaques [24, 25].

Recent research findings suggest that some novel factors may affect coagulation and fibrin clot formation [26]. In the coagulation cascade reaction, apart from the known coagulation factors, plasma kininogen can activate the single-chain urokinase and plasminogen to play an antithrombotic role. Kinins and their degradation also exhibit anticoagulant activity [27]. Elevated factor XI (FXIa) levels, increased fibrinogen carbonylation, neutrophil extracellular traps (NETs) formation, and decreased or absent antithrombin activity are associated with an elevated risk of intravascular thrombosis [26]. FXI is a plasma glycoprotein with a molecular weight of about 160 kDa and comprises two identical polypeptide chains linked by disulfide bonds to form a dimer. FXI is activated by activated FXII (FXIIa) or by the negative charge on the surface of thrombin, converting to FXIa. FXIa activates FX in the presence of FVIIIa, calcium ions, and phospholipids, leading to thrombin production and ultimately causing fibrinogen to transform into the fibrin polymer, forming a thrombus. Increased carbonylated fibrinogen promotes the formation of fibrin thrombus clots, increases the fragility of the thrombus, and reduces the strength of the clot [28]. NETs, which are composed of DNA, histones, and antimicrobial proteins, myeloperoxidase, and elastase, form an extracellular mesh structure, serving as a scaffold for thrombosis. NETs are associated with the development and progression of coronary artery disease and acute myocardial infarction. The negative charge on the surface of NETs activates FXII, initiating the intrinsic coagulation pathway [26]. Antithrombin is the major endogenous anticoagulant (serine protease inhibitor) in humans, consisting of 432 amino acids, with the main function of inactivating thrombin and FXa, and to a lesser extent, FXIIa, FXIa, and FIXa, and plays an antithrombotic role. Antithrombin dysfunction (antithrombin deficiency) can increase the risk of intravascular thrombosis. Profilin-1 is a crucial protein for cell biology, and is a protein with a molecular weight of 14 to 17 kDa. The pathogenesis of atherosclerosis, coronary thrombosis, and STEMI is perhaps associated with profilin-1 functions, including regulating aerobic energy generation, mitochondrial fission, apoptosis, reactive oxygen species (ROS) generation, and neutralization. Profilin-1 was overexpressed in patients with STEMI and correlated with the extent of myocardial infarction size [29, 30].

## 3. New Biomarker Predicting Coronary Thrombosis and No Reflow

Fibroblast growth factor-21 (FGF-21) is also recognized as a new biomarker predicting coronary no reflow in patients with STEMI. The results reported by Koprulu *et al*. [31] demonstrate that compared with STEMI patients without the no reflow phenomenon, STEMI patients with no reflow had considerably higher FGF-21 levels, and ≥92.2 pg/mL, 87% sensitivity and 88% specificity levels were found for FGF-21 in predicting the event of coronary no reflow during the PCI procedure. NETs are associated with thrombosis, ischemia, atherosclerosis, and acute myocardial infarction [32]. The product of the neutrophil/lymphocyte ratio multiplied by the CRP/albumin ratio is called the inflammatory prognostic index (IPI), which is regarded as a novel marker. The IPI in the no reflow group (n = 178) was higher than that in the reflow group (n = 1363; *p* < 0.05) [17]. The other factors, such as protein carbonylation, circulating lipopolysaccharide, and zonulin, are associated with ischemic stroke, myocardial infarction, pulmonary embolism, and cardiovascular death [22]; these biomarkers play a role as predictors of the risk of thrombosis and STEMI with heavy thrombus burden.

## 4. Identification and Judgment Criteria of Intracoronary Thrombus Burden

**Fig. 1.**
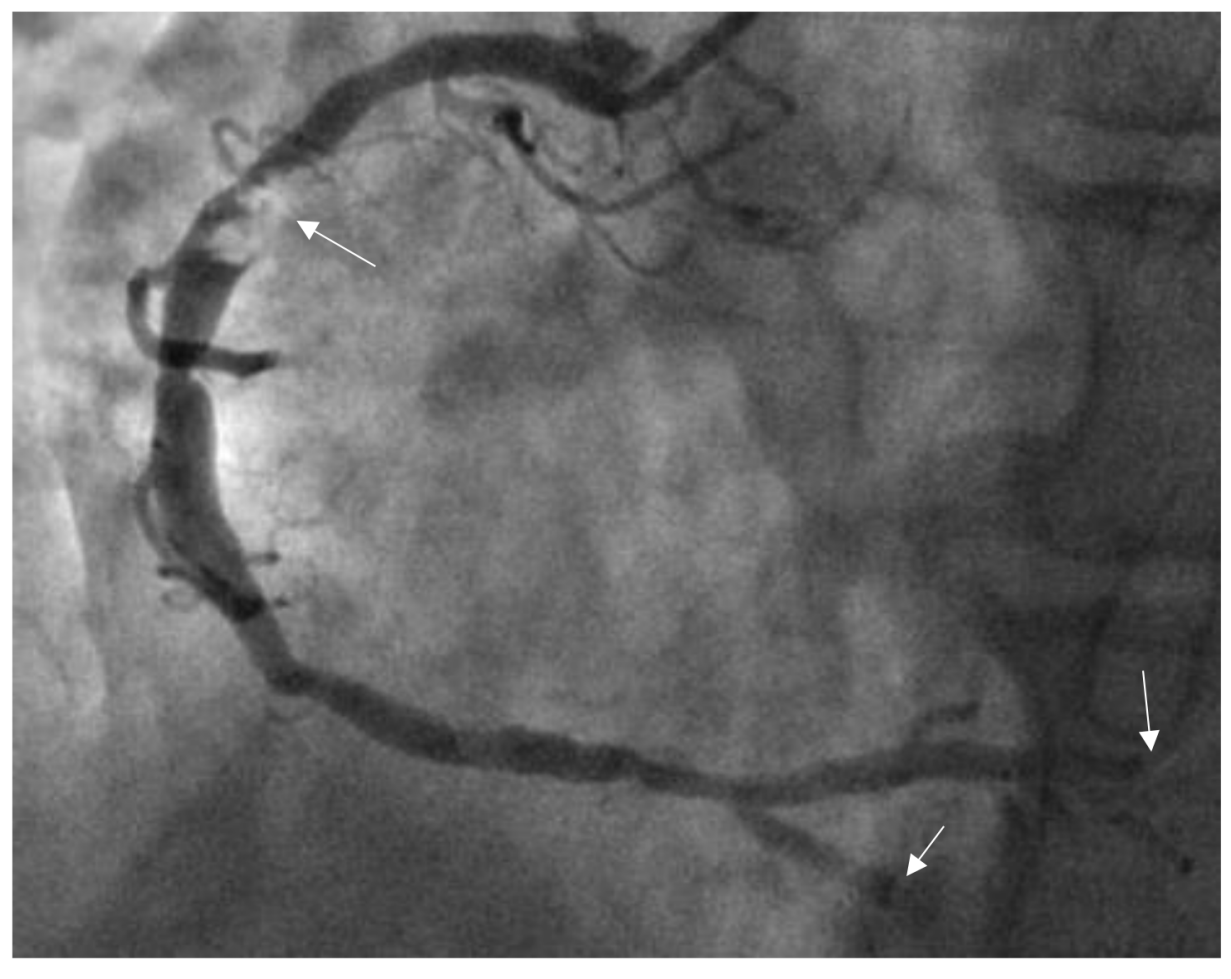
**Thrombus images in the right coronary artery (RCA)**. The patient was a male aged 46 years and was admitted to our hospital due to chest pain for 4 hours. Electrocardiogram (ECG) showed 0.50–0.60 mV ST-segment elevations in leads Ⅱ, Ⅲ, and aVF. CAG showed a 90% stenotic lesion with thrombus images in the proximal RCA (indicated by the arrow), and a thrombus score of 4 points (the patient was diagnosed and treated by the authors at the Affiliated Hospital, Beihua University).

**Fig. 2.**
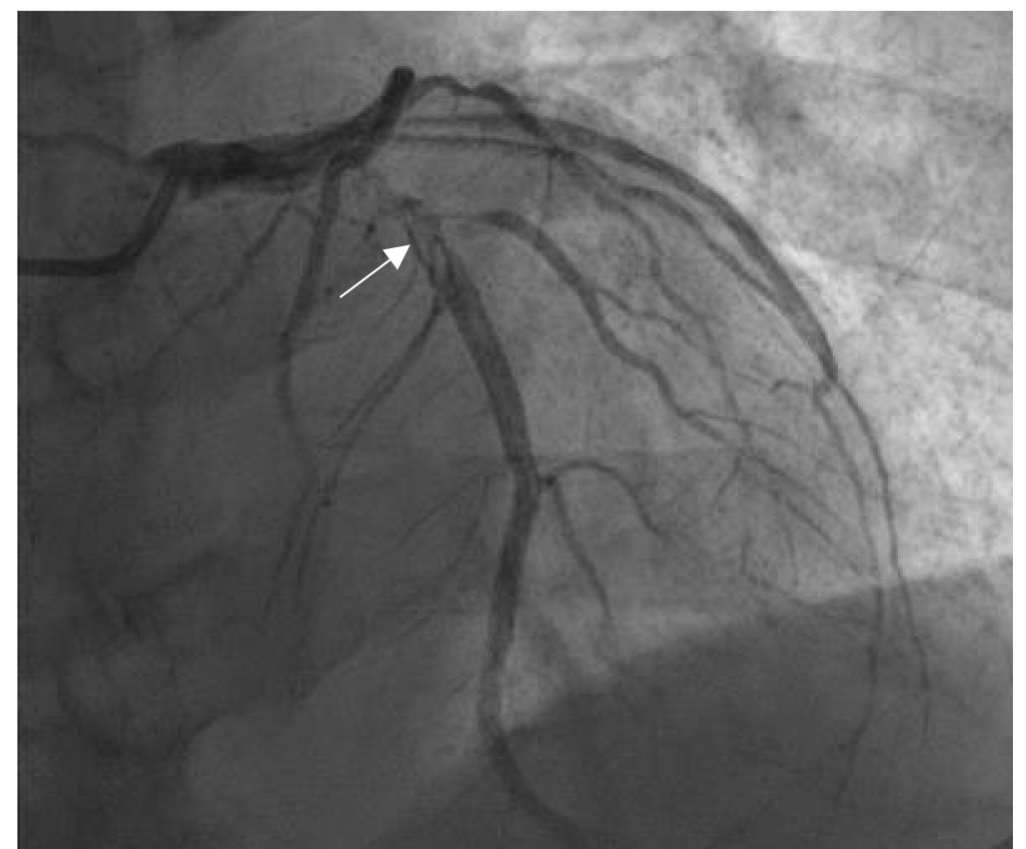
**Thrombus images in the proximal left anterior descending coronary artery (LAD)**. The patient was a female aged 58 years, who suffered from chest pain for 6 hours. The ECG showed ST-segment elevations of 0.3–0.4 mV on admission in the V2–V5 leads. CAG showed the stenosis (90% in diameter) and thrombus images (arrow indication) in 6–7 segments of LAD (thrombus score 4 points). The author diagnosed and treated the patient in the Jilin People’s Hospital in Jilin, China.

Intracoronary thrombosis is a process that accompanies the occurrence and development of STEMI. Identification of intracoronary thrombus and atherothrombosis is central to the treatment of STEMI. There are various ways to identify intracoronary thrombus burden, including invasive coronary angiography (CAG), intravascular ultrasonic imaging (IVUS), angioscopy, and optical coherence tomography (OCT). Despite the advancements in imaging technology, CAG remains the predominant imaging technique for identifying thrombosis in patients with STEMI, as it provides critical information on the extent of thrombus burden. Imaging techniques with specific scoring systems can assess the amount of intracoronary thrombus burden according to the manifestations of thrombosis in CAG. The image of thrombosis in the coronary artery in CAG is manifested as obvious filling defects or irregularly shaped blurred shadows or floating objects in the lumen of the coronary vessel, which can be seen at multiple angles of CAG and persist for numerous cardiac cycles (Figs. 1,2). The international unified thrombolysis in myocardial infarction (TIMI) thrombus score is the quantitative judgment standard for intracoronary thrombus burden [33]. That is: 0 point is no thrombus; 1 point defined as a fuzzy shadow; 2 points are defined as thrombus imaging in which the length is less than half the blood vessel diameter; 3 points is defined as the presence of blood clots where the length is 1/2–2 times the vascular diameter; 4 points are presented when the diameter and the length of certain blood clots are greater than twice the vascular diameter; 5 points refer a complete occlusion of the vessel of coronary artery. Coronary thrombus scores ≥4 were considered a high thrombus burden, and ≤3 were defined as a low thrombus burden. More recently developed invasive intravascular imaging approaches (including angioscopy, IVUS, and OCT) can provide more direct visualization of coronary atheroma and unstable plaque (vulnerable plaque) and improve the sensitivity of thrombus detection. However, these intravascular imaging techniques are not routinely employed owing to the heightened complexity of the primary percutaneous coronary intervention (PPCI) procedure and the associated economic costs. These intravascular imaging methods can evaluate residual thrombus in the infarction-related artery (IRA) after the thrombus aspiration and PCI procedure. Computerized tomography (CT), magnetic resonance imaging (MRI), and positron emission tomography (PET) can also be used to discover thrombosis in a coronary artery, and have become more widely accessible. However, noninvasive angiographic techniques cannot be used to evaluate the intracoronary thrombus of the IRA when STEMI is treated with emergency PCI; those techniques can also be used to assess the efficacy of antithrombotic therapies or the selection of interventional strategies in routine clinical practice [34, 35].

## 5. Slow Flow or No Reflow in the IRA

### 5.1 Discrimination of Coronary Artery Blood Flow Velocity

Given the pathogenesis of STEMI, the occurrence and development of STEMI are always accompanied by thrombosis in the coronary artery. Intracoronary artery thrombosis can obstruct the coronary artery lumen, which leads to the phenomena of slow flow/no reflow, and acute ischemic injury and necrosis in the myocardium, resulting, clinically, in STEMI. The composition of these thrombi significantly influences the prognosis and selection of therapeutic strategies for STEMI patients. The TIMI flow grade in the IRA is evaluated before and after performing percutaneous transluminal coronary angioplasty (PTCA), and/or thrombus aspiration and stenting. The TIMI flow grade classification standard in the IRA [2, 36] is defined as follows: grade 0 refers to the absence of forward blood flow; grade 1 refers to the imaging of micro blood flow, but the contrast agent cannot reach the distal vessels of coronary arteries; grade 2 is defined as the imaging of partial blood flow perfusion, but the contrast agent cannot reach the distal vessels within three cardiac cycles; grade 3 is normal forward blood flow perfusion and the contrast agent can reach the distal vessels within three cardiac cycles [33]. Meanwhile, ST-segment elevation can be seen on the electrocardiogram when slow blood flow (TIMI flow grade 2) or no blood flow (TIMI flow grade 0–1) occurs in the IRA. The phenomenon of slow blood flow or no reflow refers to the situation where PTCA opens the IRA; however, the antegrade blood flow in the IRA cannot reach TIMI grade 3, remaining at TIMI grade 0–2, and the ischemic myocardial tissue cannot return to normal perfusion, that is, there is no antegrade blood flow in the IRA. The causes of this phenomenon include distal coronary embolism, ischemic myocardial injury, endothelial cell injury or dysfunction, reperfusion injury, microvascular dysfunction, etc. Intracoronary high thrombus burden lesions are the primary factors contributing to slow flow or no reflow in the IRA PCI [36, 37]. For STEMI patients with high thrombus burden, pre-dilation with ballooning may result in thrombus embolization, which increases the incidence of slow flow/no reflow events [38]. The slow flow/no reflow events may aggravate myocardial damage, significantly reduce the benefits from emergency PCI for STEMI, and lead to poor outcomes for some patients [39].

In clinical practice, it has also been found that some patients exhibit coronary slow blood flow and angina symptoms; those patients have no coronary artery lesions and coronary thrombosis confirmed by coronary angiography. The pathogenesis of this slow flow is entirely different from that of slow flow in STEMI patients. Generally, this slow flow is believed to be associated with microcirculatory dysfunction, increased microvascular resistance, reduced coronary perfusion pressure, anatomical factors of the coronary arteries (such as coronary artery dilation, larger diameter, and tortuous vessels), high blood viscosity, hyperlipidemia, hyperglycemia, etc. Inflammatory factors may play an important role in the pathogenesis of the coronary slow flow phenomenon (CSFP) in patients without coronary vessel stenotic lesions and thrombus. A newly developed inflammatory marker, pan-immune–inflammation value (PIV), is an independent predictor factor for CSFP, and has a higher predictive value for the CSFP than other inflammatory markers, such as the neutrophil–lymphocyte ratio, platelet–lymphocyte ratio, and systemic immune–inflammation index [40]. However, the value of these inflammatory factors in predicting slow blood flow during PCI requires further research.

### 5.2 Management of Slow Flow/No Reflow

In clinical practice, antithrombotic drugs and coronary vasodilators are administered via a guide catheter to prevent and treat conditions characterized by slow flow or no reflow. The medications used to inject into the coronary artery include platelet glycoprotein IIb/IIIa (GP IIb/IIIa) receptor inhibitors (such as tirofiban, which inhibits platelet aggregation and blocks thrombosis, significantly improving the prognosis of patients with thrombosis), verapamil (which can hinder how the calcium ions are transferred into the vascular smooth muscle cells, dilate the coronary artery, relieve and prevent micro-coronary vessel spasms), adenosine (which dilates coronary vessels, reduces the number of neutrophils in the ischemic area, inhibits the production of free radicals from neutrophils, and maintains the integrity of the endothelium in the ischemic area), and sodium nitroprusside (which directly forms nitric oxide and rapidly becomes bradykinin to dilate the coronary artery). Based on our practical experience, when the drugs mentioned above are ineffective, injecting a small amount of epinephrine (20 µg–50 µg/time) into the coronary artery can effectively improve the slow blood flow/no reflow. Epinephrine has a significant effect on blood pressure and heart rate. According to our clinical experience, after the epinephrine is injected into the coronary artery, the heart rate rapidly increases, the myocardial contractility is enhanced, the blood pressure rises sharply, the coronary perfusion pressure increases, and the coronary blood flow accelerates, which rapidly improves the perfusion of the myocardium. The effect of accelerating the heart rate and elevating the blood pressure lasts for 2–3 min, and then the blood pressure and heart rate return to a stable level. Six articles have previously reported [41] that intracoronary epinephrine successfully restored coronary flow in over 90% of patients in managing the no reflow phenomenon following PCI. No malignant ventricular arrhythmias were observed in the patients treated with intracoronary epinephrine. Intracoronary injection of nicardipine (a calcium channel blocker) can relax coronary vascular smooth muscle, increase coronary artery blood flow velocity, and does not affect blood pressure and heart rate. Nicardipine can also increase microcirculatory perfusion and improve the slow flow/no reflow conditions. Tokdil *et al*. [42] reported that tirofiban (GP IIb/IIIa inhibitor) was infused into the distal infarct-related coronary artery in patients with STEMI and high thrombus burden or slow flow/no reflow, which showed microvascular obstruction and infarct size (that was assessed by cardiac magnetic resonance) in the distal intracoronary infusion group were significantly lower than in the systemic intravenous infusion group (*p* < 0.05). The results of a meta-analysis reported by Shah *et al*. [43] demonstrated that prasugrel is equally effective compared to ticagrelor in preventing myocardial infarction. The incidence of stent thrombosis, stroke, or major bleeding and all-cause mortality in the prasugrel group (n = 32,759) was not significantly different compared with the ticagrelor group (n = 61,831). Thrombolytic agents, including urokinase, recombinant human urokinase, recombinant tissue plasminogen activator, and pro-urokinase, effectively dissolve distal microthrombi, which improves the slow flow of IRA. The rate of TIMI grade 3 and major adverse cardiovascular events (MACEs) in the intracoronary pro-urokinase combined with the low-pressure balloon pre-dilatation group (n = 90) was significantly higher (*p* < 0.05) than in the intracoronary pro-urokinase without low-pressure balloon pre-dilatation group (n = 89). This result demonstrates that the used thrombolytic agents, followed by the opening of the infarction-related arteries, are associated with better outcomes [44]. Intracoronary tirofiban is related to a role in decreasing the infarct size. Basuoni *et al*. [45] reported the findings of a study on 100 patients with large anterior STEMI and high thrombus burden, demonstrating that decreased infarction size at 30 days was associated with intracoronary tirofiban, as assessed by cardiac MRI. In summary, intracoronary-administered thrombolytic and antiplatelet agents significantly improved myocardial perfusion and MACEs in patients with STEMI and large intracoronary thrombosis burden [46, 47].

## 6. Management Strategies for Intracoronary High Thrombus Burden

### 6.1 Thrombus Aspiration

**Fig. 3.**
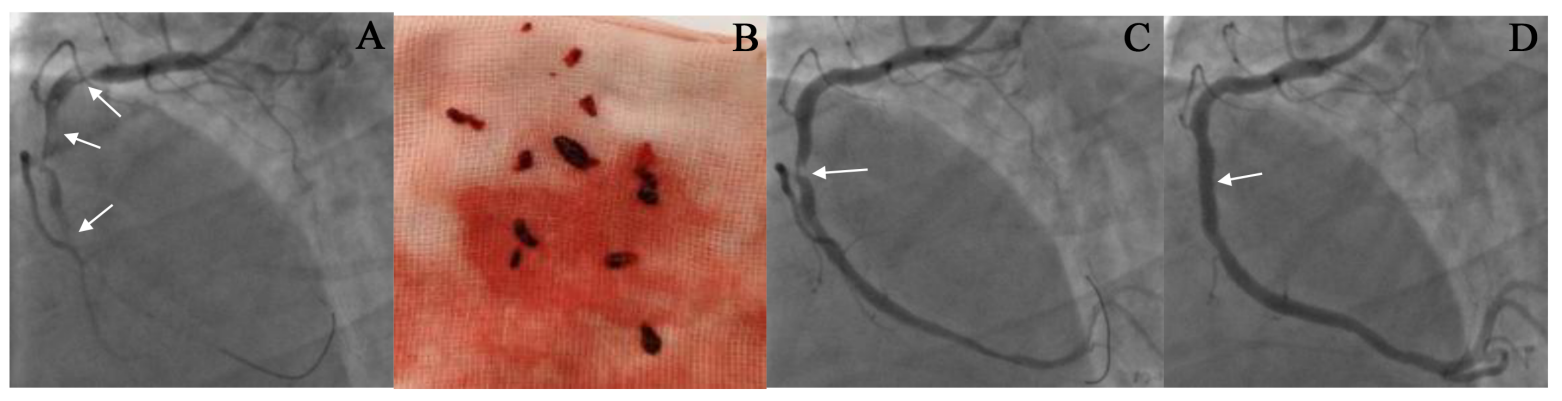
**The result of thrombus aspiration in the right coronary artery (RCA)**. (A,C,D) CAG images before and after thrombus aspiration. A 53-year-old male patient was admitted to our hospital due to chest pain for 8 hours. The ECG showed an ST-segment elevation of 0.4–0.6 mV in the inferior leads. CAG revealed complete occlusion in the proximal RCA with a large thrombus burden, as indicated by the arrow in (A). Following the administration of thrombolytic drugs, thrombus aspiration was performed seven times to remove the clot (See (B)). After the thrombus aspiration, CAG showed a residual stenosis of 90% in the RCA (as shown by the arrow in (C)), with a TIMI flow grade of 3. The condition of the patient improved significantly after the procedure. A stent was implanted at the location of the stenosis (as shown by the arrow in (D)). Repeated CAG showed that the RCA was patent, and the blood flow was TIMI grade 3 (See (D)).

A specially designed aspiration catheter is inserted into the thrombosis site of the IRA, and negative pressure suction is performed to remove the thrombus from the body (Fig. 3). The thrombus aspiration includes a manual and mechanical approach; however, the effects of the two approaches are similar [48, 49, 50]. A recent meta-analysis of small, randomized trials and observational studies indicated that thrombus aspiration helps reduce distal embolization and has a preventive effect on slow flow/no reflow. The findings from large randomized clinical trials (such as TASTE and (ThrOmbecTomy with PCI *vs*. PCI ALone in patients with STEMI) TOTAL studies) failed to show a mortality benefit and have raised concerns about their efficacy and safety, leading to updated guidelines that do not recommend routine use during primary PCI [51]. Moreover, no significant differences in clinical outcomes have been reported between the thrombus aspiration group (manual or mechanical thrombus aspiration devices) and the non-thrombus aspiration group.

Recent studies have shown that thrombus aspiration, whether manual or mechanical, can improve coronary blood flow and reduce the distal vessel embolic events of coronary arteries compared to non-thrombus aspiration strategies [48, 52]. The meta-analysis results indicate that using aspiration thrombectomy devices during PCI could reduce the phenomenon of distal embolization and improve myocardial perfusion; however, no improvement was noted in the survival rate and prognosis of patients with STEMI [53]. The results of the TOTAL study [54] differ from the above outcomes. In the TOTAL study, 10,064 cases were randomly divided into the aspiration thrombectomy group (n = 5035) and the PCI-alone group (n = 5029). The results showed that the incidence of endpoint events at 1 year (cardiovascular deaths, recurrent non-fatal myocardial infarction, cardiogenic shock, and heart failure with New York Heart Association (NYHA) class IV) was not significantly different (8.1% *vs*. 8.3%; *p* > 0.05) between the two groups. The incidence of stroke at 1 year was significantly higher in the thrombectomy group than in the PCI-alone group (1.2% *vs.* 0.7%; *p* < 0.05). Recent studies have confirmed aspiration thrombectomy benefits patients with high thrombus burden STEMI. The results of a comprehensive network meta-analysis on a total of 43 randomized trials (n = 26,682) demonstrated the risk of MACEs (odds ratio, OR 0.86), mortality (OR 0.85), myocardial infarction (OR 0.6) and target vessel revascularization (OR 0.86) in the aspiration thrombectomy group were lower than in the PCI-alone group [55]. Zajdel *et al*. [56] demonstrated that the coronary microvascular obstruction, myocardial infarct size, and left ventricular remodeling in the thrombus aspiration + PCI group were lower than those in the PCI-alone group at 6 months (*p* < 0.05), which was confirmed by MRI. Moreover, Mathews *et al*. [57] demonstrated that sustained mechanical aspiration before PCI in ACS patients with high thrombus burden was associated with high rates of thrombus removal, flow restoration, and normal myocardial perfusion confirmed by CAG. Jeon *et al*. [58] also demonstrated a higher rate of myocardial blush grade 3, higher ST-segment resolution, and a lower incidence of microvascular obstruction in the successful thrombus aspiration group (n = 533) compared with the failed thrombus aspiration group (n = 279). Alkhalil *et al*. [59] proved that thrombus aspiration strategies benefit STEMI patients with a large thrombus burden.

Based on the studies mentioned above, although different outcomes were observed, most results suggest that selective thrombus aspiration is associated with better clinical outcomes. The various outcomes of clinical randomized trials are due to the fact that the intracoronary thrombosis burden in STEMI patients was not considered a grouping factor. The results of the existing studies have proven that thrombus aspiration can benefit patients with high thrombus burden (Fig. 2) and improve the success rate of PCI [60], but cannot benefit those with low thrombus burden.

Updated guidelines suggest that routine thrombus aspiration during PPCI for STEMI patients is not generally beneficial and has been downgraded to a class III recommendation. The description in the ESC guidelines is “Routine thrombus aspiration is not recommended, but in cases of large residual thrombus burden after opening the vessel with a guide wire or a balloon, thrombus aspiration may be considered” [61, 62]. In view of the above, the following principled recommendations are in favor of selective thrombus aspiration for STEMI patients: (1) Routine thrombus aspiration is not recommended during primary PCI; (2) the patients with a high thrombus burden in IRA and TIMI flow of 0 to 1 grade, and with a larger vessel diameter in IRA, may benefit from thrombus aspiration (IIb recommendation); (3) when the manual aspiration for patients with high thrombosis burden is ineffective the mechanical thrombus aspiration may be used. These recommendations have practical guiding significance and have been widely recognized. Selective thrombus aspiration benefits patients with high thrombus burden [2].

### 6.2 Percutaneous Intracoronary Thrombolysis

Percutaneous coronary artery thrombolysis involves injecting thrombolytic agents into the IRA of STEMI patients through CAG catheters or PCI guide catheters, enabling the thrombolytic agents to directly contact and rapidly dissolve the thrombus, thereby reopening the IRA. Thrombolytic therapy is mainly applicable to patients with a TIMI flow grade of 1–2 in the IRA. In patients with a TIMI flow grade of 0, it is difficult for a thrombolytic agent to reach the thrombus or to come into direct contact with thrombi, meaning the effect of thrombolysis remains uncertain. CAG can observe the impact of IRA recanalization and thrombus reduction within 20–30 minutes after the thrombolytic agents are injected. Intracoronary thrombolysis can also dissolve residual thrombi after aspiration thrombectomy, which is beneficial in reducing the occurrence of slow flow/no reflow. The thrombolytic agents include urokinase (200,000 u are injected into the coronary arteries), alteplase (15–30 mg are injected into the coronary arteries), reteplase (10 MU plus 10 MU intravenously in two separate injections, with a 30-minute interval), prourokinase (intracoronary injection of 100 mg, which can be repeated), and so on [62, 63, 64]. For the use of thrombolytic drugs in coronary arteries, please refer to the drug’s instruction manual.

### 6.3 PTCA and Deferred Stenting

PTCA is an effective method for opening an IRA vessel. Utilizing a PTCA balloon, under adequate anticoagulation therapy (heparinization), dilates the occluded coronary artery to restore forward blood flow, with reported success rates of up to 96.3%. When the blood flow is restored to TIMI flow grade 2–3, the intracoronary thrombus will slowly dissolve spontaneously (automatic thrombolysis). It has been clinically proven that the intracoronary thrombus in patients with STEMI will disappear or be significantly reduced after the antithrombotic (antiplatelet plus anticoagulant) treatment is performed for one week following PTCA. At this stage, the severity of the fixed stenotic lesions in the IRA became clear, and the implantation of stents (known as deferred stenting) can effectively reduce the complications of slow flow/no reflow and distal embolism, and improve myocardial perfusion and the left ventricular ejection fraction [65].

However, it has been debated whether delayed stenting is beneficial to STEMI. Liu *et al*. [2] reported that in STEMI patients with high intracoronary thrombosis burden, deferred stenting could significantly reduce the incidence of slow flow/no reflow and distal embolization, and improve the rate of target lesion vessel patency compared to immediate stenting. A total of 208 geriatric patients (aged ≥80 years) with STEMI and a high thrombus burden (thrombus score ≥4) in the IRA were categorized into a deferred stenting group (n = 132, with stenting delayed by 7–8 days) and an immediate stenting group (n = 76). The results of the study demonstrated that in the deferred stenting group and the immediate stenting group, the rate of TIMI flow grade 3 was 98.48% *vs.* 84.21% (*p* < 0.01), and the rate of distal embolization was 3.03% *vs.* 36.84% (*p* < 0.01); the rate of myocardial blush grade (MBG) 3 was 98.48% *vs.* 76.32% (*p* < 0.01), the rate of MACEs 9.32% *vs.* 20.27% (*p* < 0.05), and left ventricular ejection fraction (LVEF) was 0.60 ± 0.05 *vs.* 0.58 ± 0.05 (*p* < 0.01). These results suggest that the patient outcomes in the deferred stenting are better than those in the immediate stenting, which is consistent with the results reported by Carrick *et al*. [66].

However, the DANAMI 3-DEFER trial yielded contrasting outcomes [67], whereby 1215 STEMI patients were randomly divided into an immediate stenting group (n = 612) and a delayed stenting group (n = 603; delayed for 48 hours), with an average follow-up of 42 months (33–49 months) in both groups. The results of the study showed that there was no significant difference in the incidence of MACEs (including all-cause mortality, hospitalization for heart failure, recurrent non-fatal myocardial infarction, and target vessel revascularization between the two groups (*p* = 0.92), which indicated that delayed stenting was not beneficial to STEMI patients. However, the study results showed a significant improvement in LVEF in the delayed stenting group (*p* < 0.05) compared with the immediate stenting group; it cannot be considered that deferred stenting is not beneficial for all STEMI patients. In the study design, because the thrombus burden score in the IRA is not considered a grouping factor, the results and conclusion in the randomized controlled trial study differ from the outcomes of other observed studies. If patients with a high thrombus burden are further divided, potentially diverse outcomes could emerge by assigning the intracoronary thrombus burden in patients with STEMI to the immediate and delayed stenting groups for subgroup analysis. Therefore, it cannot be conclusively stated that deferred stenting is universally beneficial for all STEMI patients. Consequently, it cannot be denied that selective deferred stenting may benefit patients with a high thrombus burden, as suggested by clinical studies. A delay of less than 48 hours may not be sufficient for effective spontaneous thrombolysis in IRA, as indicated by studies [2].

### 6.4 Reasonable Delay Time for Deferred Stenting

The debate continues on the optimal interval between the initial PTCA that opens an IRA and restores TIMI flow grade 3, and the subsequent PCI with stenting. Liu *et al*. [2] reported that the ideal deferral time is 7 to 8 days. In 208 geriatric patients with STEMI and a high thrombus burden, the deferred stenting group (delay 7–8 days) was associated with shorter stent implantation in length, larger stent implanted in diameter, a lower incidence of distal embolism in the IRA, higher rates of TIMI flow grade 3 and myocardial blush grade 3, increased LVET and lower MACEs compared to those in the immediate stenting group (*p* < 0.05). Those results are similar to the study outcomes reported by Magdy *et al*. [68] and Pradhan *et al*. [69]. The study results reported by Magdy *et al*. [68] revealed that the deferred stenting (delayed 7 days) was associated with a higher rate of TIMI flow grade 3 and MBG grade 3, and a lower incidence rate of no reflow and MACEs at 6 months, compared with the immediate stenting. Pradhan *et al*. [69] reported that the deferred PCI stenting (delayed 5–7 days) for STEMI with high thrombus burden can reduce the thrombus burden, improve blood flow and myocardial blush grade, and prevent unnecessary stent implantation. The outcomes of the sub-study from the DANAMI-3-DEFER trial [70] revealed that the deferred stenting (549 STEMI patients with large thrombus burden) was associated with lower incidences of slow flow/no reflow and distal embolization (*p* < 0.05) compared with immediate stenting (n = 656).

These studies found that clinical outcomes were improved in deferred stenting compared to immediate stenting when the delay was around 7 days, as evidenced by various clinical analyses and meta-analyses. The good outcomes may be because the thrombus naturally dissolves, the vasospasm is relieved, TIMI blood flow improves, and the risk of slow flow/no reflow is reduced during the 7–8-day period of antithrombotic therapy. The extent of the lesion and the diameter of the IRA can be demonstrated after the thrombus autolysis or disappearance during a 7–8-day delay, which avoids the implantation of small-diameter stents and long stents, and unnecessary stent implantation, which will theoretically reduce the long-term target vessel revascularization rate [2].

### 6.5 Primary Stenting

Most STEMI patients should undergo primary stenting after PTCA. Delayed stenting should be used only in patients with the intracoronary high thrombus burden, those at high risk of slow flow/no reflow, or those with hemodynamic and electrocardiographic instability. According to the updated guidelines, PPCI with stenting is the preferred treatment for patients with STEMI, particularly when initiated within 12 hours from symptom onset to balloon inflation [61]. The results from previous studies showed that emergency PPCI for STEMI patients under the support of hypothermia may reduce mortality [71, 72]. Nevertheless, the latest research results from a meta-analysis of randomized controlled trials reported by Mhanna *et al*. [73] revealed that therapeutic hypothermia did not benefit patients more than the standard PCI in 706 patients with STEMI (in 10 randomized controlled trials). The application of hypothermia in conjunction with PCI has increased the complexity and cost of the procedure, without conferring any additional clinical benefits [72, 73], thereby hindering its widespread adoption.

### 6.6 Drug Administration May Reduce Thrombus Burden 

Drugs that reduce thrombus burden include heparinization and platelet glycoprotein (GP) IIb/IIIa receptor inhibitors, calcium ion antagonists, vasodilators, etc. Those drugs have a good effect on reducing thrombosis and preventing slow flow/no reflow. For STEMI patients with an intracoronary high thrombus burden, statins and antiplatelet new medicines used before PCI help to reduce the thrombus burden. The GP IIb/IIIa receptor inhibitors (such as tirofiban, ticagrelor, and prasugrel) prevent platelet activation and aggregation to block thrombosis and distal embolization. Tirofiban can be injected into the coronary artery to increase the local drug concentration in the IRA, which helps the drug enter the thrombus, relaxes the thrombus, reduces the thrombus burden, and reduces circulation embolism [74]. The dual antiplatelet therapy (DAPT) of aspirin and the P2Y12 receptor inhibitor (clopidogrel, prasugrel, or ticagrelor) in patients with ACS who undergo PCI was recommended by clinical guidelines. Ticagrelor and prasugrel may be more effective and safer than clopidogrel in ACS patients. The effect of ticagrelor on inhibiting platelet aggregation is faster and stronger than that of clopidogrel. Studies have shown that ticagrelor reduces the incidence of cardiovascular death and myocardial infarction compared with the clopidogrel group [75].

## 7. Limitations

Several limitations should be considered. The contents described in this paper are enriched by the personal experiences of the author and closely align with the latest published research results, showing our understanding and approach to the cardiovascular field. While most of our insights are grounded in the outcomes of published studies, further research is needed to confirm and enrich the practical knowledge.

## 8. Conclusion

In summary, the occurrence and development of STEMI are accompanied by thrombosis and thrombus accumulation, which results in coronary artery occlusion. The main pathogenesis of STEMI is the existence of unstable atherosclerotic plaques (known as vulnerable plaques) on the coronary artery wall. The vulnerable plaque rupture triggers the coagulation cascade, leading to the formation and progression of thrombi. During PCI for STEMI patients, due to a large thrombus burden in the coronary artery, slow flow/no reflow may occur, endangering the lives of patients and causing PCI failure. For STEMI patients with high thrombus burden, thrombus aspiration during PCI removes intracoronary thrombi effectively, reduces the incidence rate of slow flow/no reflow events, and improves the perfusion level of myocardial tissue, which is beneficial to protecting heart function and enhancing the outcomes of STEMI patients with high thrombus burden. The antithrombotic and thrombolytic drugs can be used as an adjunct to PCI. For patients with a lower thrombus burden, it is reasonable to treat with PPCI and stenting.

## Abbreviations

ACS, Acute coronary syndrome; ADP, Adenosine diphosphate; AT, Aspiration thrombectomy; CA, Coronary atherosclerosis; CAG, Coronary angiography; CC, Cholesterol crystals; CHD, Coronary heart disease; CSFP, Coronary slow flow phenomenon; DATP, Dual antiplatelet therapy; ENTs, Neutrophil extracellular traps; ESC, Esterified cholesterol; FAs, Fibroatheromas; FDF, Fibroblast growth factor; FRC, Free cholesterol; FXII, Factor XII; GP IIb/IIIa, glycoprotein IIb/IIIa; HDL, High-density lipoprotein; IPI, Inflammatory prognostic index; IRA, Infarct-related artery; IVUS, Intravascular ultrasonic imaging; kDa, Kilodalton; LAD, Left anterior descending coronary artery; LDL-c, Low-density lipoprotein cholesterol; LVEF, Left ventricular ejection fraction; MACE, Major Adverse Cardiovascular Events; MBG, Myocardial blush grade; MRI; Magnetic reasonance imaging; NLR, NOD-like receptor; NLRP3, NLR family pyrin domain containing protein 3; NOD, Nucleotide oligomerization domain; NSTEMI, Non-ST-segment elevation myocardial infarction; NYHA, New York Heart Association; OCT, Optical coherence tomography; PCI, Percutaneous coronary intervention; PPCI, Primary percutaneous coronary intervention; PTCA, Percutaneous transluminal coronary angioplasty; PVI, Pan-immune-inflammation value; RCA, Right coronary artery; RCT, Randomized Control Trial; ROS, Reactive oxygen species; STEMI, ST-segment elevation myocardial infarction; TCFAs, Thin-cap fibroatheromas; TIMI, Thrombolysis in Myocardial Infarction.
